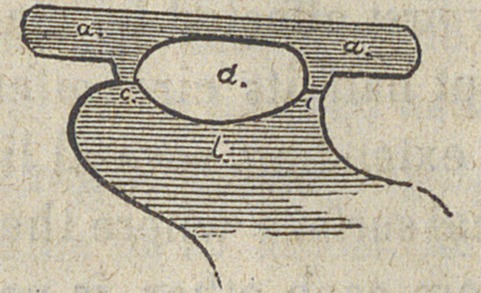# Clasps

**Published:** 1853-10

**Authors:** B. T. Whitney

**Affiliations:** Buffalo, N. Y.


### *Clasps, by* DR. B. T. WHITNEY, *Buffalo, N. Y.*

The adaptation of partial sets of artificial teeth, in a manner so as to secure the greatest utility and comfort to the patient, and at the same time, produce the least unfavorable results, calls for, not only general dental reading, but much observation and good judgment.

The mere operation of mounting one or more teeth upon a plate, and adapting them to the mouth so as to answer the name of *teeth* is a simple work of art, and is easily accomplished by any *tyro* in dental mechanics. But every person who has given this operation close attention for a twelve month, has, doubtless, seen sad havoc made with sound natural teeth by the clasps used for securing these pieces. Cases are constantly multiplying, where one plate after another has been inserted, and one plate after another destroyed, until in a few years, the person is bereft of the last of his dental family. Indeed so disastrous is the result, that many careful and conscientious dentists have hesitated, or actually refused to supply the loss of some particular teeth, advising that it is better to endure the absence of one or two than have them replaced for a short time at the expense of others that are more valuable

In the hands of our best dentists, and notwithstanding the greatest care in the adaptation of the plate, it too frequently occurs that the tooth upon which the wearings are made soon begins to soften, and in a short time is wholly destroyed. This may be the work of a few months only, or of several years, according to the form of the clasp or its adaptation; the kind, or plurality of metal used; or the proportion of lime in the composition of the tooth.

The prescribed limits of this article compel me only to speak of the manner of adapting clasps, so as to prevent the loss of, or do the least injury to the natural teeth. To more clearly demonstrate the point aimed at, and impress its importance on the mind of the reader, it may be well to notice the different modes of attaching small artificial pieces, and some peculiarities in the anatomy of the tooth selected for their support.

In the earlier periods of dental practice, silken ligatures, and thin gold or silver wire were used in securing small blocks of artificial teeth, which were at first simply blocks of ivory or bone, carved so as to fit the gums and represent the teeth. This block was perforated at either end or through the center, so as to receive the silken or metalic ligature, which was then secured by a turn around the adjoining natural teeth. Of the two, as well as those to be mentioned hereafter, the silken attachment, though it gave less support to the piece, caused less injury to the natural teeth—it did not denude the enamel, it formed a less recepticle for the deposit and retention of foreign matter, beside the necessity of frequently renewing it, securing partial cleanlines.

Then came the great improvement of mounting the crown of human teeth; and a little later, those made of porcelain upon a gold base, but still secured in the mouth in the same way —soon followed the farther improvement of soldering a strip of gold to either end of the plate, fitted so as to encircle the adjoining teeth in place of the ligature. These gold bands were made very narrow, sometimes a small half round wire, and pressed beneath the margin of the gum, fitting snugly around the neck of the tooth so as to be concealed. The dentine, at this point, being poorly protected by enamel, the tooth was sure soon to receive irreparable injury by the denudation of the tooth, the retention of foreign matter which soon acidulates and unites with the lime of the tooth, and irritation of the adjoining soft parts, which are separated from the tooth to give place to the clasp.

Later in the march of improvement came the broader plates, giving more stability to the base, and a gradual widening of the clasp until finally they nearly cover the depth of the crown of the tooth. This is the mode of general practice at the present day with our best dentists, and is far preferable to anything that preceded it.

Suction plates have been used, as a sure gurantee against the destructive effects of the clasp, and often with admirable success. The plan is ingenious and highly commendable: but generally patients object to this mode of mounting one, or a few teeth, from the heavy expense attending it, and its encumbering the mouth too much ; besides, it cannot, in all cases, be relied upon, and, in but few, will it give the stability and firmness so much desired.

Small pieces are sometimes attached by making the bearings simply on the palatal side of the remaining teeth. In some cases this may be admissable, but it is liable to serious objections. The bearing is made upon the very part of the tooth most liable to injury from clasps, and, unless the plate is nicely fitted and springs into its place, so as to make its principal bearing at the neck of the tooth, or above the central and rounded part of the crown, it will be too easily displaced, and, by its long continued pressure, is likely to force outward the natural teeth. This mode is sometimes resorted to to avoid seperating the natural teeth, and sometimes to conceal the clasps from view

It is seldom necessary to use the file to give room for a clasp; as generally the exercise of good judgment in the selection of the proper tooth to be used as the support of the artificial piece, without regard to the expense of the plate or labor of fitting, will enable us to adjust the clasp in the manner hereafter to be described, so as to give firmness to the piece.

As the ligature, the wire, and the light narrow clasps are now considered among the things that were, I have only to make a few reflections upon the use and effect of the broad clasps, and give some suggestions and experience in the mode of applying them, so as to obviate, to some extent, at least, the injury to the natural teeth.

It is all important that every dentist confining himself as well to the mechanical as the surgical department of practice, should well understand the anatomy and physiology of organs he is called upon to treat, and the agents that may destroy or injure them; the best preventative as well as curative means; and in all his operations upon the mouth, keep an eye to the *future* health and beauty of the natural teeth, rather than the *present* adaptation of partial substitutes.

From the anatomical structure of the teeth, it is obvious that long continued contact with any foreign or corroding agent must be attended with injurious results; soonest where the enamel is the weakest, and least where it is the thickest—or rather, that this protective covering to the softer substance of the teeth resists the action of foreign substances, other things being equal in proportion to its thickness. The tooth is always smaller at the neck or margin of the gum, where the enamel is thinner than at the center or upper part of the crown, so that in using the ordinary wide clasp, the metal comes in contact only with the tooth at its larger portion; leaving a space, greater or less in proportion to the shape of the tooth near its neck. This space only serves as a receptacle for the accumulation of foreign matter, that absorbs freely of oxigen, as atmospheric air passes through the mouth, soon decomposes, generating acid that is surely destructive to the tooth. All dentists of much observation, know that the point most liable to injury and usually first effected by clasps, is on the lingual side, embracing about half its circumference, and from the margin of the gums, extending several lines up the crown of the tooth. The labial surface where the ends of the clasp are left some distance from each other, is never the seat of decay or injury. Now, how are we to save the lingual side also from injury? There is but one way, and that is to expose as much of the tooth as possible to its native element—the cleansing effects of the saliva, friction of the tongue, &c. This can be accomplished without weakening the clasps, or detracting from the stability of the plate, in the following manner:—The strip of gold to be used for the clasp is as wide as the length of the crown will receive, and in length, so as to embrace only so much as is really necessary for the support of the piece. Fit the plate intended for the base or support of the artificial teeth, accurately to the neck of the tooth, so as to encircle the lingual half, or as nearly as possible, but leave a space of from one to two lines between them. The clasp is accurately fitted to the tooth, then with the plate, held firmly in its place, mark upon the clasp at the points of the plate at the posterior and anterior approximal sides of the tooth, and cut out a semi-circular piece from the clasp, between these marks, leaving but a small portion to come in contact with the plate at each point, just enough to solder them firmly together. The clasp, after cutting out this semi-circular piece, if straitened out is represented by fig. 1

At *a* the clasp is left but about two lines in width—*b b* is to be soldered to the base. I then proceed to solder the pieces at one point, *b* only, and place the piece in the mouth to see that the position of the clasp is right. This enables me to make any little change in the fitting and proper adjustment desired before both points are made fast. When all is completed the clasp and plate are nearly represented in fig. 2

*a a* the clasps, *b* the base. These two pieces are soldered together at *c c*—leaving by the open space *d*, the lingual side of the crown of the tooth, the point as we have seen, so liable to decay or injury, almost entirely free. The point *c c* is the only part that comes in contact with the neck of the tooth, and this is so small that it is more easily kept clean, affording less space for the deposit of perishable and injurious agents, while the bearing is upon the more prominent portion of the tooth.

From my own practice, and observing many plates that have been worn for four years, I am persuaded that this is the best means of adapting clasps so as to cause the least injury.

BUFFALO, N. Y., Sept. 5, 1853.

## Figures and Tables

**Figure f1:**
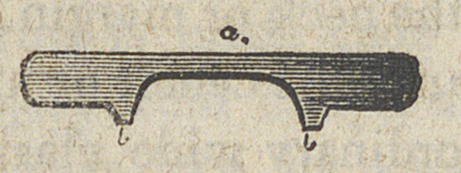


**Figure f2:**